# Tribological Evaluation of Polyether Ether Ketone (PEEK) Nanocomposite Coatings Reinforced with Ceria—Effect of Composition, Load, Speed, Counterface, and UV Exposure

**DOI:** 10.3390/polym17111487

**Published:** 2025-05-27

**Authors:** Amal A. Seenath, Mirza Murtuza Ali Baig, Abdul Samad Mohammed

**Affiliations:** 1 Mechanical Engineering Department, King Fahd University of Petroleum & Minerals, Dhahran 31261, Saudi Arabia; g202203660@kfupm.edu.sa (A.A.S.); mmurtuza@kfupm.edu.sa (M.M.A.B.); 2 Interdisciplinary Research Centre for Advanced Materials, King Fahd University of Petroleum & Minerals, Dhahran 31261, Saudi Arabia

**Keywords:** PEEK, ceria, nanocomposite coatings, wear, friction

## Abstract

Ceria nanofillers were incorporated into PEEK coatings at concentrations of 0.5, 1.5, and 3 wt% and applied to mild steel samples using an electrostatic spraying technique. The tribological performance of these coatings was assessed under various loads and sliding speeds. XRD, FTIR, and microhardness tests were conducted to characterize the chemical and mechanical properties of the coatings. The 1.5 wt% ceria-reinforced PEEK coating outperformed the pristine PEEK and other concentrations in terms of wear resistance. The counterface material did not affect the wear resistance of the optimized PEEK/1.5 wt% ceria nanocomposite coating, which also demonstrated superior wear resistance after UV exposure as compared to that of pristine PEEK coatings.

## 1. Introduction

Polymeric coatings, with their inherent adaptability, affordability, and excellent properties, have emerged as potential alternatives among the wide range of materials investigated for tribological applications. As a result, polymer and polymer composite coatings have been extensively utilized for the protection of metallic components against wear across a range of conditions. The application of these polymer coatings has been constrained by issues such as limited strength, inadequate load-bearing capability, poor stability at low temperatures, and challenges with water absorption.

Polyether ether ketone (PEEK) is a high-performance semi-crystalline thermoplastic polymer known for its exceptional combination of properties. It exhibits excellent thermal stability, superior chemical resistance, remarkable mechanical strength, and reasonably good tribological characteristics, making it suitable for demanding engineering applications [1,2,3]. Moreover, PEEK’s biocompatibility and resistance to sterilization processes make it an attractive choice for medical implants and devices, further highlighting its significance in tribology [4,5,6]. Furthermore, it demonstrates outstanding heat resistance, withstanding temperatures up to 250 °C [7,8]. Notably, they exhibit exceptional abrasion and scratch resistance. These characteristics position PEEK coatings as optimal choices for environments, demanding high-temperature performance and chemical resistance, such as those encountered in oil production, chemical manufacturing, and aerospace sectors [9,10,11,12]. Despite its remarkable attributes, pristine PEEK exhibits certain limitations when subjected to severe tribological conditions. Compared to other polymers like ultra-high molecular weight polyethylene (UHMWPE) and polytetrafluoroethylene (PTFE), which are known for their excellent biocompatibility and excellent tribological properties, it shows a relatively high wear rate and a coefficient of friction (COF) ranging from 0.30 to 0.38 [13]. Because of their low coefficients of friction, which vary from 0.05 to 0.08 and from 0.09 to 0.12, respectively, polymers like UHMWPE and PTFE have been the subject of much research [14,15,16]. However, the poor thermal stability of UHMWPE on account of its low melting point, as well as the higher wear rates of PTFE, limit their use in harsh working conditions under high loads and speeds, making PEEK an excellent candidate for coatings in demanding tribological applications. To address the issues of higher wear rate and friction coefficient in PEEK, one approach is the incorporation of nanofillers into the PEEK matrix to develop nanocomposite coatings.

The incorporation of nanoparticles into polymeric matrices is a revolutionary development in surface engineering, providing a flexible method for strengthening materials against tribological deterioration. By precisely selecting and dispersing nanoparticles throughout the polymer matrix, the tribological, thermal, and mechanical properties of the nanocomposites can be effectively tailored. The selection of reinforcements is guided by the specific application requirements. In a study by Cao et al. [17], tantalum nanoparticles were added to PEEK to produce a composite coating for bone repair applications. Tantalum was found to be a biocompatible metal with outstanding strength and resistance to corrosion [18,19]. Similarly, many researchers developed PEEK composites with different types of fillers, including Teflon (PTFE) [20,21], silicon carbide (SiC) [22,23], alumina (Al_2_O_3_) [24], titanium dioxide (TiO_2_) [25], silicon dioxide (SiO_2_) [26], carbon nanotubes (CNTs) [27], and carbon fiber (CF) [28,29].

Hence, it is necessary to select a suitable filler that will enhance the properties of the PEEK coatings. The primary objective is to discover a solution that can enhance its implementation across various industrial applications. By utilizing the exceptional thermal characteristics of PEEK in combination with the unique properties of carefully chosen fillers, the resulting composite coatings become well-suited for demanding applications, such as the high-speed, high-temperature environments encountered in components like bearings. One such filler that has not been explored and has the potential to fulfill the above requirements is cerium dioxide/ceria (CeO_2_).

Cerium dioxide is a versatile rare earth metal compound that finds usage in a wide range of biological and engineering fields. As a rare earth metal compound, ceria exhibits remarkable chemical resistance, outstanding thermal stability, and exceptional mechanical strength, making it ideal for use in demanding industrial environments [30,31]. Moreover, ceria nanoparticles have also received significant attention in both the cosmetic and materials science fields for their exceptional ultraviolet (UV)-protective properties. In sunscreens, ceria is valued for its ability to absorb and scatter UV radiation, effectively shielding the skin from harmful UV rays that can lead to sunburn, skin aging, and increased risk of skin cancer [32,33,34]. Ceria’s UV-blocking capabilities and its antioxidant properties are particularly beneficial in environments like the aerospace and automotive industries, where materials are often exposed to harsh sunlight and fluctuating temperatures [35,36]. All these properties, long with ceria’s high hardness and wear resistance behavior, make it compatible with applications involving heavy loads and high-speed operation, such as in the manufacturing of bearings, gears, and other mechanical components subjected to extreme forces [37,38,39]. Nevertheless, a survey of the available research literature indicates that no studies on PEEK-ceria nanocomposite coatings for tribological purposes are currently available. Hence, this study aims to develop and evaluate this nanocomposite coating system’s mechanical and tribological properties. In this study, tribological tests will be performed to evaluate the coefficient of friction, as well as the effect of the counterpart on the wear tests using different counterfaces on the developed coatings. In addition, tribological tests will also be conducted to evaluate the effect of UV exposure on the wear performance of the developed coatings, focusing on changes in tribological behavior before and after UV exposure.

## 2. Experimental Procedures

### 2.1. Materials

**Figure 1:**
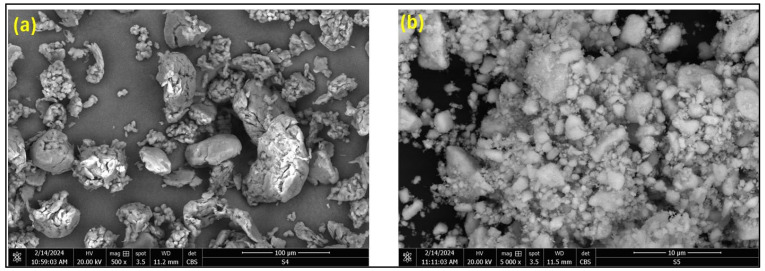
SEM micrographs of (**a**) PEEK and (**b**) nano ceria particles.

Polyether ether ketone (PEEK) powder, procured from Goodfellow Cambridge Corp (Cambridge, UK), was used as the matrix with a mean particle size of 50 µm and a density of 1.30 g/cm^3^. Cerium dioxide (CeO_2_), obtained from Sigma-Aldrich (Merck Group, USA), served as the reinforcement, featuring a mean particle size of 50 nm and a density of 7.13 g/cm^3^. A mild steel disc (Ø = 25.4 mm; thickness = 6 mm) was used as a substrate. Figure 1 illustrates the morphology and size characteristics of the selected matrix and filler particles.

### 2.2. Preparation of Nanocomposite Powders

PEEK/ceria nanocomposite powder was synthesized by magnetic stirring in conjunction with the sonication process. A probe sonicator (Sonics, Vibracell, USA) was used to sonicate a weighted quantity of ceria in 50 mL of ethanol for ten minutes at an amplitude of 30% and an on/off cycle duration of twenty/five seconds. To further distribute the ceria particles evenly, the sonicated solution with the ceria powder was magnetically agitated for two minutes at 1100 rpm using magnetic stirrers. Subsequently, a measured quantity of PEEK powder was added to the solution and magnetically stirred for one hour. The mixture was then heated in a furnace at 80 °C for 24 h to fully evaporate the ethanol, resulting in the development of PEEK/ceria nanocomposite powder for further processing.

### 2.3. Substrate Surface Preparation

Prior to the coating process, the mild steel substrates underwent surface roughening to improve bonding between the substrate and the coating. This was accomplished with a metallographic manual grinder–polisher (Mikrosize, China) equipped with 120-grit paper, achieving a final surface roughness of 2.214 ± 0.5 µm. After the grinding process, the samples were cleaned with acetone in an ultrasonic cleaner for 15 min to remove any foreign contaminants and subsequently dried using an air blower.

### 2.4. Coating Procedure

The ground and cleaned mild steel samples were preheated in an oven at 370 °C for 10 min before being removed for coating deposition using a Craftsman^®^ electrostatic spray gun (Model: 17288). The spray gun applied a powdered layer onto the grounded substrates. During the spraying process, each substrate was coated uniformly using four spray passes, with each pass lasting about 10 s, maintaining a consistent nozzle-to-substrate distance of around 30 cm. The powder-coated samples underwent a post-heat treatment in an oven at 370 °C for 30 min and were subsequently cooled to room temperature to achieve a uniform coating.

## 3. Coatings Characterizations

### 3.1. Crystallographic Evaluation

The phase transformations of the nanocomposite powders were analyzed through X-ray diffraction (XRD). The measurements were carried out at room temperature using a diffractometer (Rigaku-Ultima IV) equipped with a Cu (Kα) X-ray source. The powders were analyzed at a rate of 2θ = 10–60°, with a scan rate of 0.05°/s.

### 3.2. Hardness Evaluation of the Coating

The microhardness (Vickers) of the coatings was evaluated using an MCT^3^ tester (CSM Instruments SA, Switzerland). A 0.25 N load was applied for a dwell time of 15 s using a diamond indenter with a pyramidal shape. The indentation depth was kept below 10% of the coating thickness for each sample to reduce the substrate’s influence during indentation. The hardness values reported for each coating were the average of five different measurements [40].

### 3.3. Evaluation of the Coating Thickness and Dispersion of Ceria in the PEEK Matrix

The thickness of the coating was determined with the help of a scanning electron microscope (FE-SEM, FEI Quanta FEG 250, USA), equipped with energy-dispersive spectroscopy (EDS). The flat side of a semi-circular coated steel sample was examined for this purpose, with four measurements taken per sample, and the average thickness was reported. SEM images, combined with EDS maps, were captured to analyze the dispersion of ceria particles within the PEEK matrix. The samples were gold-coated (for around 25 s) using a Quorum (Q150R S, UK) gold coating machine to prevent the charging effect and make it conductive prior to the SEM analysis.

### 3.4. Thermogravimetric Analysis

Thermogravimetric analysis (TGA) was conducted to evaluate the thermal properties of the powder using a TGA/DSC instrument (SDT Q600, Mettler Toledo, Switzerland) in an inert nitrogen atmosphere. Samples weighing 10 ± 2 mg were heated at a steady rate of 10 °C/min, within a temperature range of 25 °C to 700 °C, while maintaining a nitrogen flow rate of 50 mL/min.

### 3.5. Ultraviolet (UV) Radiation Tests

The UV resistance of the coatings on steel panels (Q235) was evaluated using the QUV Accelerated Weathering Tester (QUV/Spray Gen 4, UK). The test was carried out according to ASTM G154-23 standards. Each cycle was set to 8 h UV irradiation (UVA-340+) and water spray, followed by 2 h condensation, for a total of 500 h. Temperature and humidity were set to 60 ± 3 °C and 50 ± 5% RH, respectively.

### 3.6. Fourier Transform Infrared (FTIR) Spectroscopy

The FTIR spectroscopy of the coated samples was analyzed using the DTGS KBr disc method, employing a Nicolet FTIR iS10 system (Thermo Scientific, USA), covering the wavelength range from 4000 to 500 cm^−1^.

### 3.7. Tribological Characterization

The wear tests were conducted at room temperature using a ball-on-disc setup on a UMT-3 tribometer (Bruker, USA). A 6.3 mm diameter alumina ball served as the counterface, which was cleaned with acetone and dried using an air blower prior to the testing. To examine the formation of any transfer film or scratches, the counterface ball was inspected using an optical microscope (MT7000, Meiji, Japan), both before and after the experiment. To investigate how various counterface materials influence the tribological properties, three additional balls—silicon nitride (Si_3_N_4_), tungsten carbide (WC), and 440C hardened steel (SS), with similar specifications to the alumina ball—were used. Each sample underwent three sets of tests, and the average values for the wear life and COF were presented.

### 3.8. Evaluation of Wear Morphology

Following the wear tests, the wear track was examined, and subsequently, the coating failure characteristics were thoroughly analyzed using an FE-SEM with EDS. To prevent surface charging during SEM analysis, a thin layer of gold coating was applied on each sample. To measure the depth of the wear track (Z) after each test, both two-dimensional (2D) and three-dimensional (3D) wear profiles were captured using a GTK-A optical profilometer (Bruker, USA).

## 4. Results and Discussion

### 4.1. XRD Analysis of the Composite Powders

**Figure 2:**
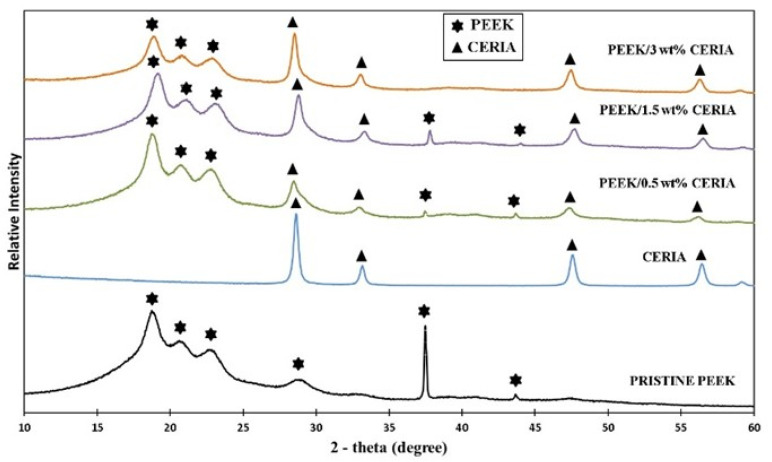
XRD patterns of pristine PEEK and PEEK reinforced with ceria at different loading conditions.

The XRD patterns for the pristine PEEK powder were obtained in the 2θ range of 10 to 60°, which shows the characteristic diffraction peaks of crystalline PEEK in its α-phase at 18.8°, 21.6°, 22.8°, and 28.7°, corresponding to (110), (111), (200), and (211) of the diffraction planes, respectively (Figure 2) [40,41]. The XRD patterns of ceria were also plotted in the 2θ range of 25 to 60°, corresponding to (111), (200), (220), and (311) of the diffraction planes to provide a detailed understanding of the intensity and shift in the XRD peaks obtained in the composites prepared for this study [42]. The X-ray diffractogram of the composite powders exhibited a pattern equivalent to that of pristine PEEK and ceria, characterized by wider and less intense peaks. Despite the extremely low ceria composition for 0.5 wt% addition, there is a small change in peak intensities suggesting possible interactions between PEEK and ceria nanoparticles, although these effects could be negligible [43,44]. More prominent peaks appeared at 2θ angles between 27 and 30° in the XRD analysis of 1.5 and 3 wt% ceria loadings. These angles correlate to the existence of ceria nanoparticles. The presence of ceria also caused similar intense peaks to be seen at 45–47° and 56–58°, respectively. A distinctive crystalline phase inside the polymer matrix is shown by the peak in the pristine PEEK’s XRD pattern, which is seen at about 37.5°. This crystalline structure of the PEEK was decreased, as seen by the reduced peak intensity at 0.5 wt% ceria. However, at 1.5 wt% ceria nanoparticles, the peak intensity increased, indicating a more uniform dispersion of the nanoparticles and potential restoration of the crystalline structure. On the other hand, at 3 wt%, the peak completely disappeared, indicating substantial changes in the crystalline structure due to nanoparticle agglomeration [45].

### 4.2. Analysis of Microhardness

**Figure 3:**
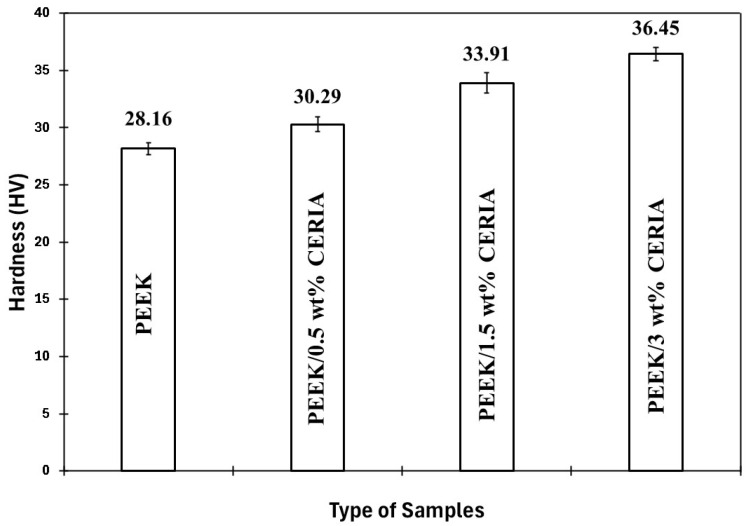
Hardness comparison of PEEK nanocomposite coatings with varying ceria loadings.

Figure 3 illustrates the variation in the hardness of the PEEK coatings with varying loadings of the ceria nanoparticles. In general, an increase in the hardness values of the coatings was observed with the addition of ceria nanoparticles. Incorporating well-dispersed nanoparticles into the parent matrix enhances the stiffness of the matrix by limiting the slippage and mobility of polymer chains, consequently resulting in an elevation in hardness values [46,47]. Here, the smaller ceria nanoparticles have larger surface area-to-volume ratios thereby enhancing interfacial interactions. Reinforcing ceria has resulted in creating a greater interfacial area between the two media, facilitating efficient stress transfer between the nanoparticles and the polymer matrix, promoting effective resistance to indentation, and increasing the microhardness.

### 4.3. Analysis of the Thickness Measurements and Distribution of Nanoparticles in the Parent Matrix

**Figure 4:**
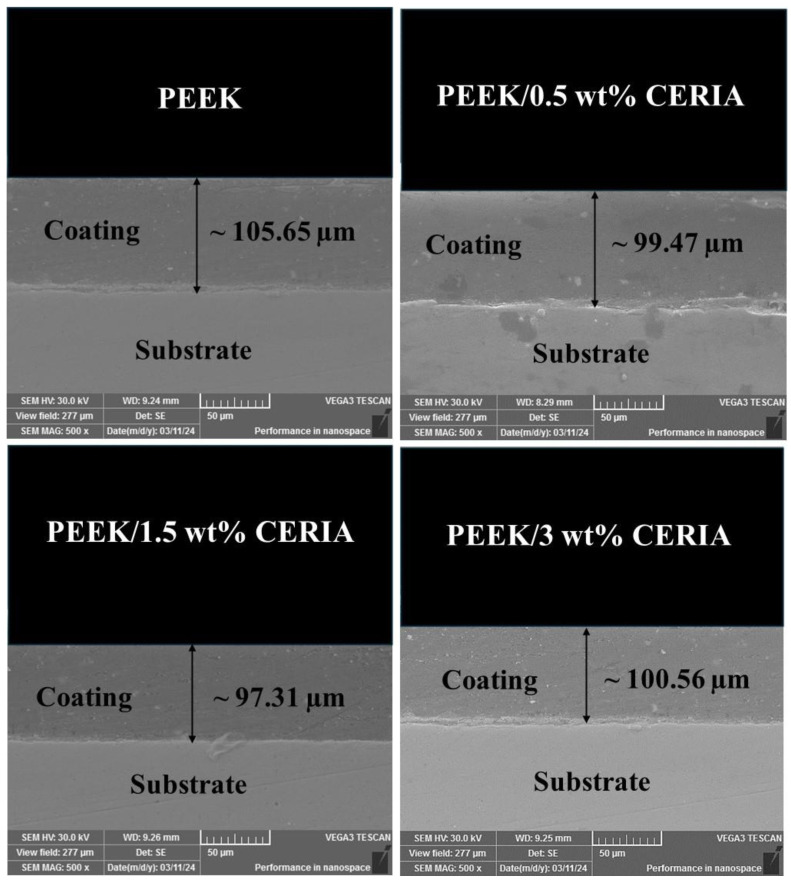
Cross-sectional SEM images of the coatings.

**Figure 5:**
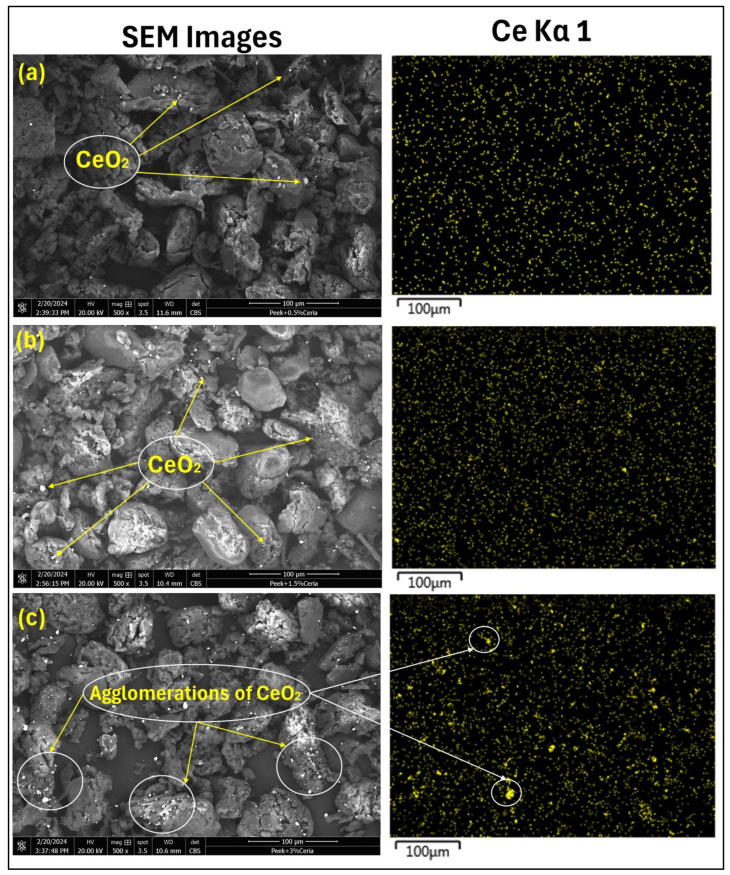
SEM images PEEK matrix reinforced with (**a**) 0.5 wt%, (**b**) 1.5 wt%, and (**c**) 3 wt% ceria loadings, along with EDS maps for cerium (Ce).

Figure 4 presents the thickness measurements of pristine PEEK and PEEK/ceria nanocomposite coatings. It was noted that incorporating the nanofillers did not significantly change the thickness values. The average value of the thickness readings of the coatings was found to be ~100 ± 5 µm. Figure 5 depicts the EDS elemental maps of cerium (Ce), which offers insights into the distribution of ceria nanoparticles within the PEEK composite coatings at varying weight percentages. At 0.5 wt%, ceria nanoparticles demonstrate a lower concentration of nanofiller throughout the polymer matrix. However, at 1.5 wt%, the overall distribution is relatively uniform, indicating the effective distribution of the ceria nanoparticles within the PEEK polymer matrix. In contrast, at 3 wt%, very pronounced agglomerations of ceria nanoparticles become evident, suggesting non-uniform distribution within the polymer matrix. Various researchers performed such observations underlying the importance of dispersion analysis of the ceria nanoparticle in their studies [48,49].

### 4.4. Thermal Stability

**Figure 6:**
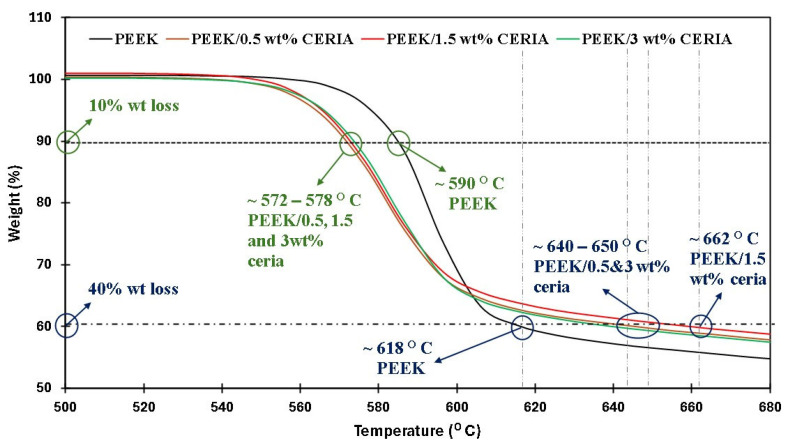
TGA thermograms of pristine PEEK and PEEK nanocomposite powders.

Figure 6 shows the TGA curves of pristine PEEK powder along with the PEEK/ceria nanocomposite powders. It is evident that the PEEK powder lost 10% of its weight at 590 °C, while the 0.5, 1.5, and 3 wt% reinforcements lost 10% of its weight at around 572–578 °C. As the temperature progressed, it can be concluded that a 40% weight reduction was observed for pristine PEEK powder at 618 °C, while the nanocomposite powders exhibited a 40% weight reduction at 641 °C, 662 °C, and 647 °C for 0.5, 1.5, and 3 wt% reinforcements, respectively.

At elevated temperatures, the presence of ceria has slowed down the decomposition process. This observation is in agreement with the TGA curves of pure PEEK, exhibiting a lower weight loss at lower temperatures, followed by a significant increment in the weight loss as the temperature progressed when compared to its composite counterparts [50]. Similar observations were also reported in ref. [51], where the TGA curve of PEEK exhibited a similar trend.

### 4.5. Tribological Results

The initial set of wear tests were conducted to determine the load-bearing capacity and the sliding speed limitation of pristine PEEK coatings deposited on mild steel substrates.

#### 4.5.1. Effect of Load and Linear Speed on the Tribological Performance of Pristine PEEK Coatings

**Figure 7:**
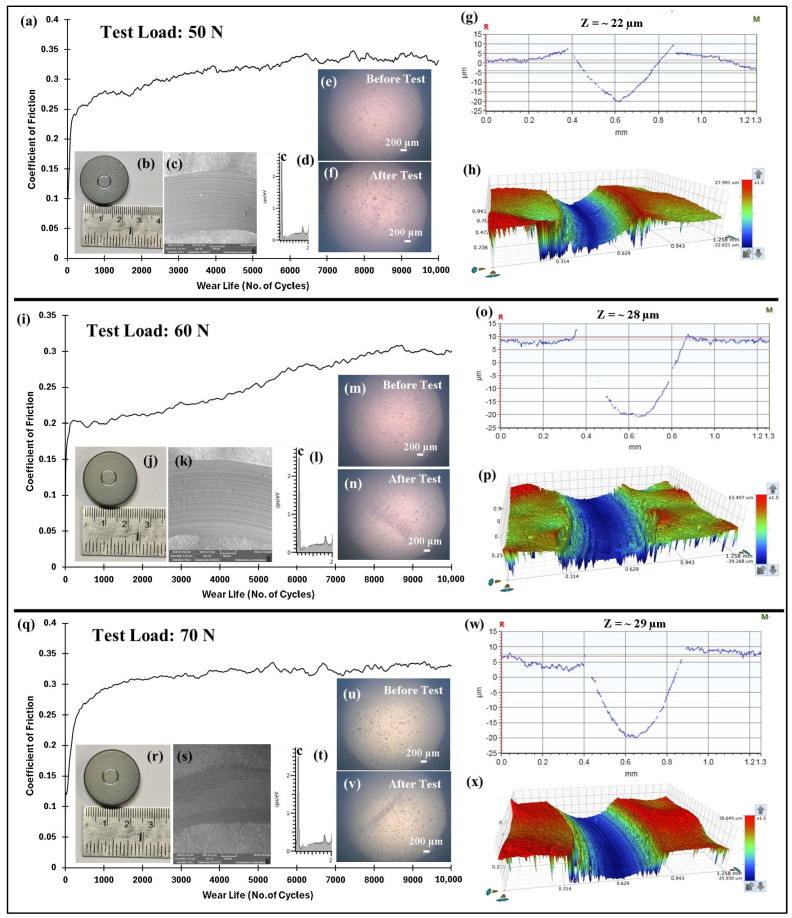
Comparison of the (**a**,**i**,**q**) frictional graph of pristine PEEK coatings, along with (**b**,**j**,**r**) wear track photographs, (**c**,**k**,**s**) SEM images and the corresponding (**d**,**l**,**t**) EDS scans, the (**e**,**m**,**u**) counter face ball images before and (**f**,**n**,**v**) after the test, the (**g**,**o**,**w**) 2D depth, and the (**h**,**g**,**p**,**x**) 3D profile images of the wear track at varying normal loads for 10,000 cycles at 0.1 m/s.

Tribological tests were conducted on pristine PEEK coatings to assess their ability to withstand applied loads. The tests were carried out under normal loads of 50, 60, and 70 N, with a steady sliding speed of 0.1 m/s (318 rpm) over 10,000 cycles, resulting in a total sliding distance of 189 m. The wear track radius was consistently fixed at 3 mm throughout the experiments. Notably, the tests were immediately stopped if the COF surpassed 0.5, indicating the possibility of metal-to-metal contact and coating failure. The frictional performance and wear track depth for normal loads of 50, 60, and 70 N are shown in Figure 7.

**Figure 8:**
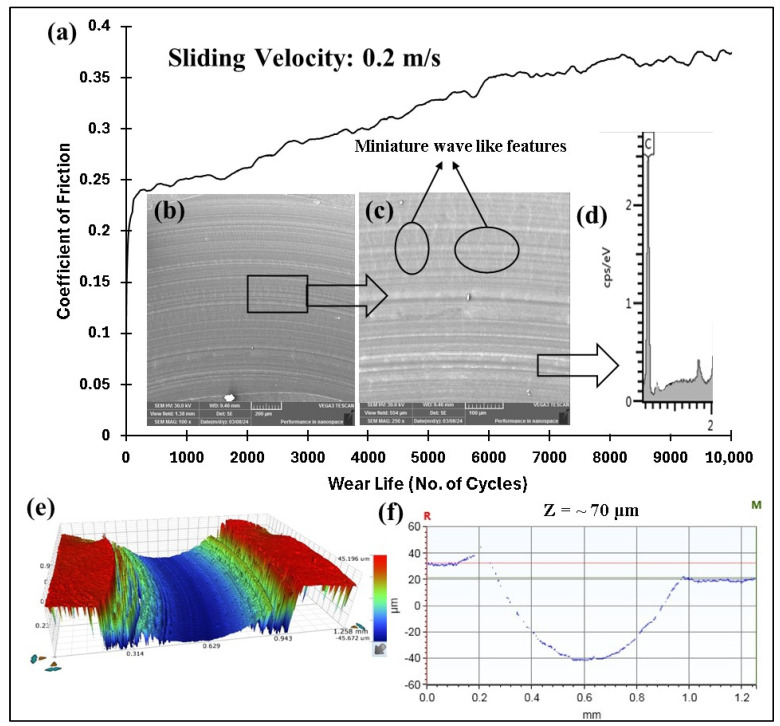
Pristine PEEK coating at a normal load of 70 N, including the (**a**) frictional graph and (**b**,**c**) SEM image, along with the (**d**) corresponding EDS scan, (**e**) 3D optical scan, and (**f**) 2D optical scan of the wear track.

**Figure 9:**
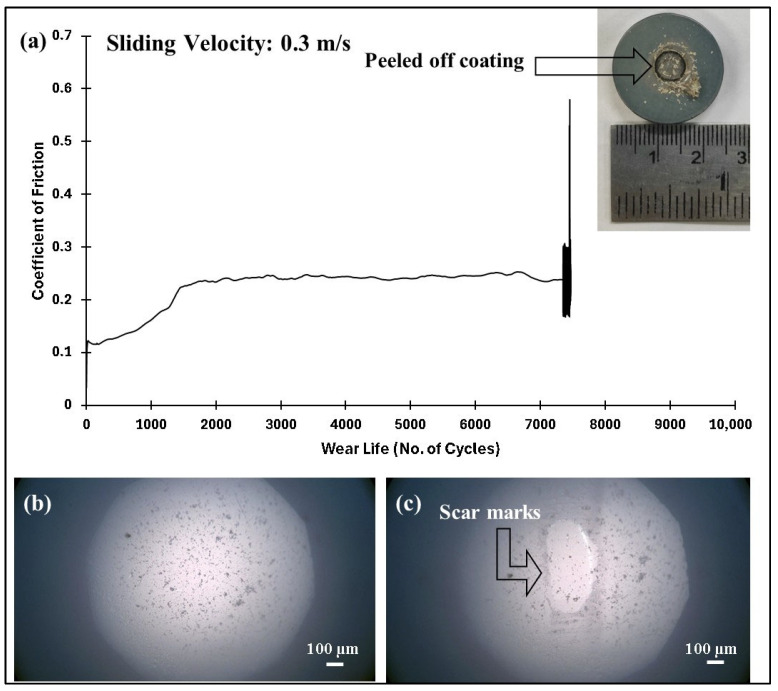
Pristine PEEK coating at a normal load of 70 N, including the (**a**) frictional graph, along with the peeled-off coating with the (**b**) ball image before and (**c**) after testing with acetone cleaning.

**Figure 10:**
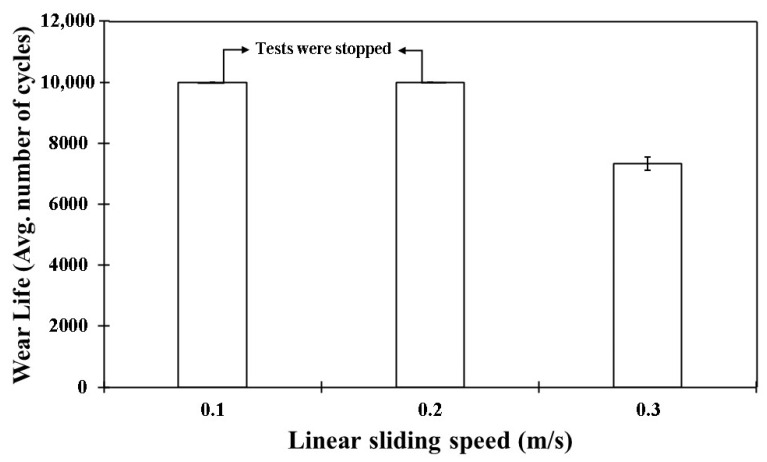
Wear life comparison for pristine PEEK coatings under a normal load of 70 N over 10,000 cycles at varying sliding speeds.

The COF graphs, combined with the wear track images and corresponding wear track depth profiles (Z), which remained below the coating thickness of ~100 µm, confirmed that the pristine PEEK coating withstood up to 10,000 cycles without failure, even as the load increased from 50 to 70 N. Additionally, it is evident that as the load increased, the wear track depth also increased. This can be attributed to the higher contact pressures, which resulted in more material being removed and deeper wear tracks. The frictional graphs also support these observations. The pristine PEEK coating appeared to reach a critical load threshold as the load was increased from 60 to 70 N, beyond which increasing the normal load did not notably alter the wear behavior at a sliding speed of 0.1 m/s. This threshold is likely due to the inherent mechanical properties of the PEEK coating, such as its wear resistance, toughness, and hardness, which allowed it to endure higher contact pressures without experiencing excessive wear. To explore how sliding speed affects the tribological performance of pristine PEEK coatings, further wear tests were conducted by incrementally increasing the sliding speed from 0.1 to 0.5 m/s, while keeping the normal load constant at 70 N. The pristine PEEK coating tested at 0.2 m/s (637 rpm) exhibited a COF of 0.35 and survived for 10,000 cycles without failure. The SEM image, along with the EDS map, confirms this (Figure 8). As the sliding speed was increased from 0.1 to 0.2 m/s, a significant increase in wear track depth (~70 µm) was observed. At these conditions, the surface experienced maximum shear stress due to the increased friction. The repetitive sliding of the counterface ball across the coating led to the appearance of small wave-like features on the wear track, primarily caused by surface fatigue resulting from cyclic stresses. These stresses, induced by the repetitive motion of the counterface ball over the softer PEEK coating, led to the formation of subsurface cracks. However, when the linear speed was further increased to 0.3 m/s (955 rpm), the coating failed after ~7500 cycles, as shown in Figure 9. It is important to note that with higher sliding speeds, the localized temperature rises due to insufficient heat dissipation time, causing the PEEK matrix to soften and leading to coating failure. This phenomenon is reflected in the TGA curves in Figure 6, where the degradation rate of PEEK increases with temperature, ultimately causing significant deterioration of material properties and coating failure at higher loads and speeds. Figure 10 summarizes the wear life comparison of the pristine PEEK coatings, clearly indicating that the maximum load and speed it could withstand for 10,000 cycles were 70 N and 0.2 m/s.

#### 4.5.2. Effect of Load and Linear Speed on the Tribological Performance of PEEK/Ceria Nanocomposite Coatings

**Figure 11:**
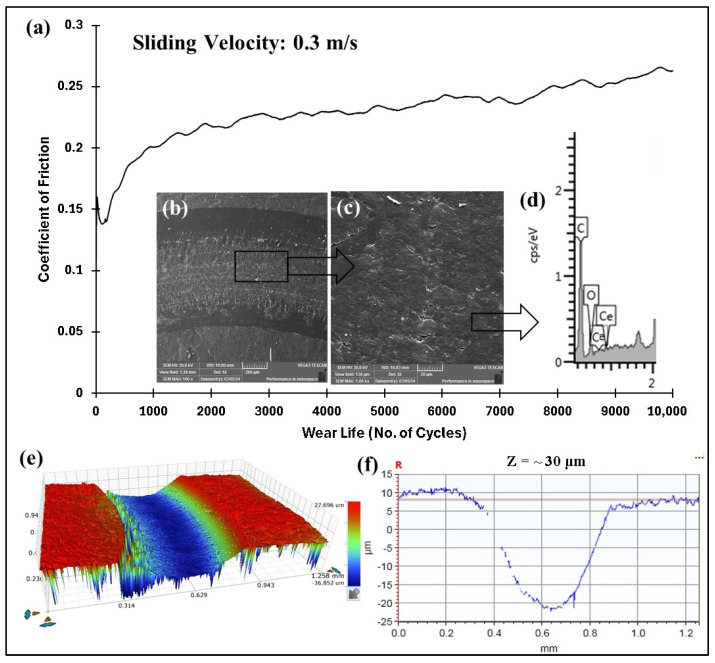
PEEK/0.5 wt% ceria coating at a normal load of 70 N, including the (**a**) frictional graph and (**b**,**c**) SEM image, along with the (**d**) corresponding EDS scan, (**e**) 3D optical scan, and (**f**) 2D optical scan of the wear track.

**Figure 12:**
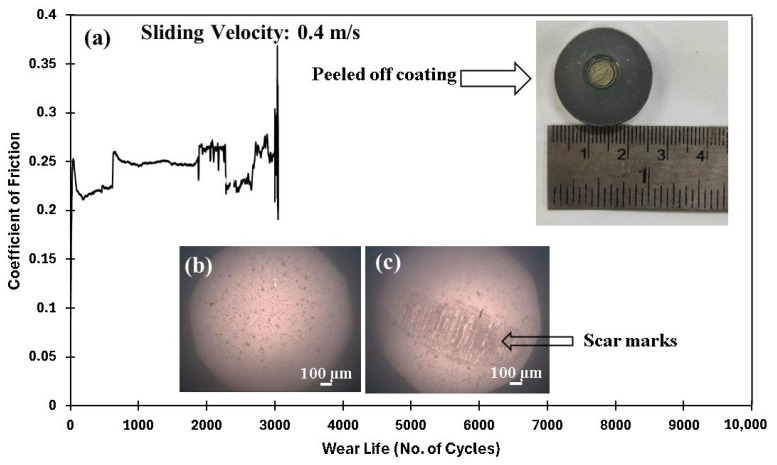
PEEK/0.5 wt% ceria coating at a normal load of 70 N, including the (**a**) frictional graph and the peeled-off coating with the (**b**) ball image before and (**c**) after testing with acetone cleaning.

After testing the pristine PEEK coatings, which failed at 70 N load and 0.3 m/s sliding speed, nanocomposite coatings with ceria loadings of 0.5, 1.5, and 3 wt% were developed, and their tribological properties were assessed under these conditions. As illustrated in Figure 11f, incorporating ceria enhanced the resistance to wear properties of the PEEK coating. Under the same load and sliding speed, the wear track depth reduces to ~30 µm, in contrast to the ~70 µm observed for the pristine PEEK coating. These enhancements can be attributed to the increased hardness of the coating achieved by incorporating 0.5 wt% ceria. However, at 70 N and 0.4 m/s, the PEEK/0.5 wt% ceria coating failed ~3100 cycles, as illustrated in Figure 12.

**Figure 13:**
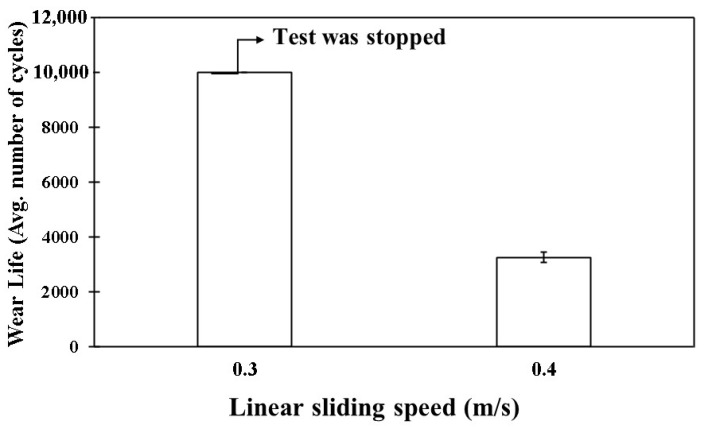
A comparative study of the wear life of PEEK/0.5 wt% ceria coatings under a normal load of 70 N over 10,000 cycles at varying sliding speeds.

As the sliding speed is increased, the relative motion between the coating and the counterface medium intensifies, resulting in increased frictional forces and contact stresses at the interface, leading to a considerable generation of heat and increased localized temperatures at the interface. Hence, the coating failure must have resulted from the reduction in the mechanical properties as a result of the degrading of the coating at these elevated temperatures, signifying the fact that 0.5 wt% of ceria was insufficient to provide the necessary reinforcement to the coating at an increased speed of 0.4 m/s. The comparative wear life of PEEK/0.5 wt% ceria coating is shown in Figure 13.

**Figure 14:**
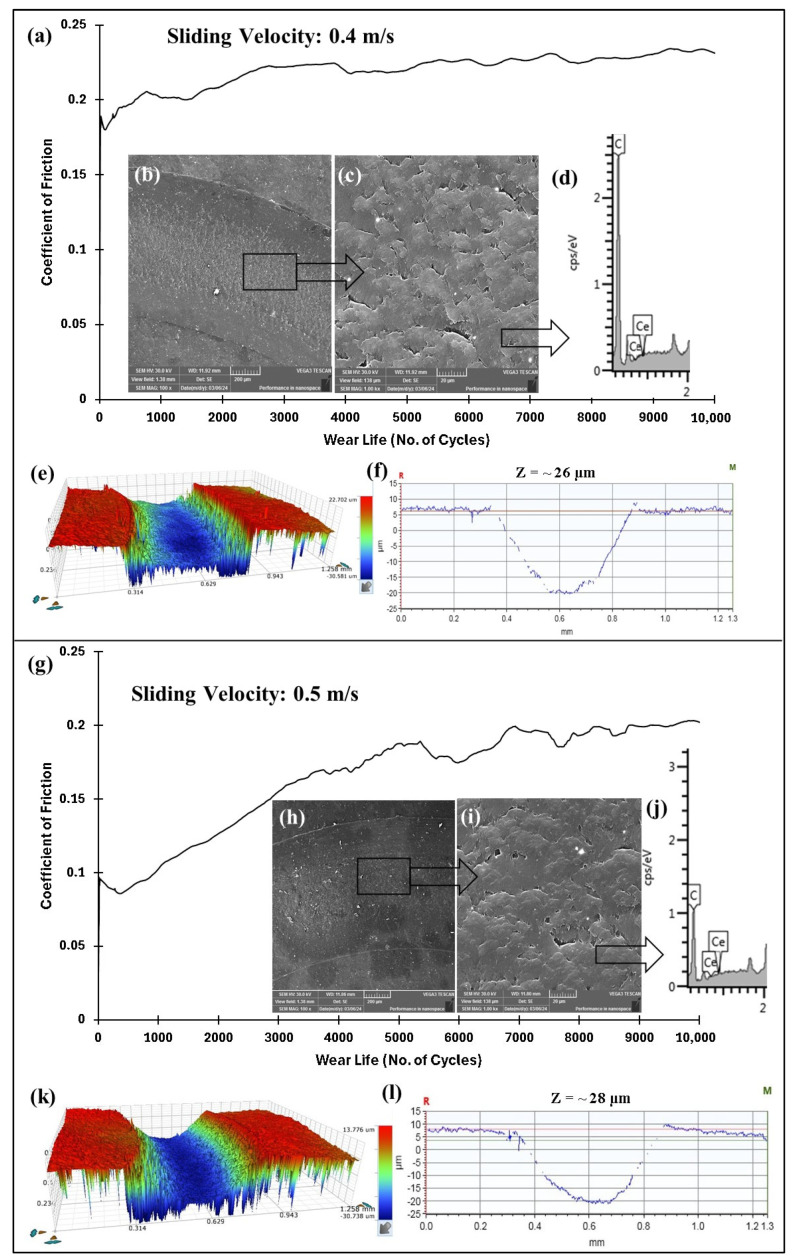
(**a**,**g**) Frictional graphs of PEEK/1.5 wt% ceria coatings, along with (**b**,**c**,**h**,**i**) SEM images with the corresponding (**d**,**j**) EDS scans, (**e**,**k**) 3D profile, and (**f**,**l**) wear track depth images of the wear track at varying sliding velocities for 10,000 cycles at 70 N.

**Figure 15:**
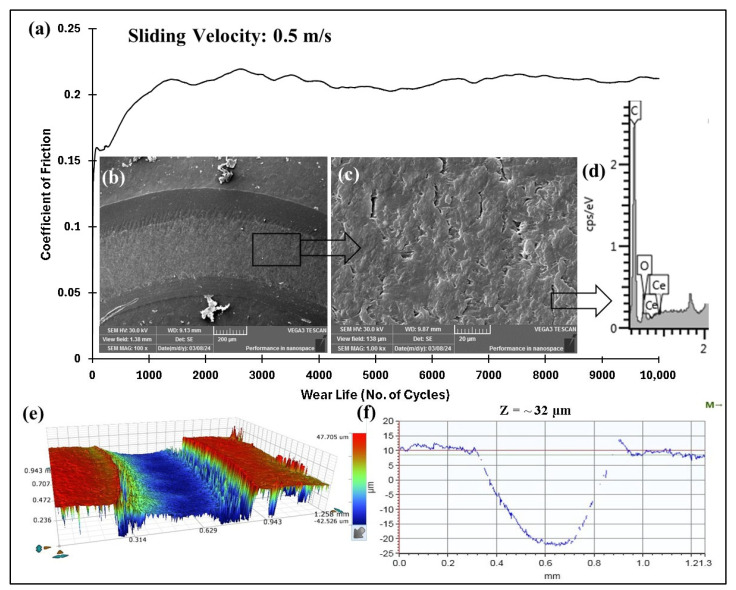
PEEK/3 wt% ceria coating at a normal load of 70 N, including the (**a**) frictional graph and (**b**,**c**) SEM images, along with the (**d**) corresponding EDS scan (**e**), 3D optical scan, and (**f**) 2D optical scan of the wear track.

As the PEEK/0.5 wt% ceria coating failed at 70 N and 0.4 m/s, the wear test was subsequently conducted on the PEEK/1.5 wt% ceria coating under the same conditions. Figure 14a–f illustrate the performance of the PEEK/1.5 wt% ceria nanocomposite coating at operating conditions of 70 N and 0.4 m/s, where it can be observed that the nanocomposite coating did not fail up to 10,000 cycles. The linear speed was then increased to 0.5 m/s, and as shown in Figure 14g–l, the PEEK/1.5 wt% ceria nanocomposite coating also sustained up to 10,000 cycles without failure. This is confirmed by the 2D profiles of the wear tracks, which show wear track depths of ~26 µm and ~28 µm, respectively, which are significantly lower than the coating thickness. It is also evident from the EDS scans that the coating did not fail as there were no noticeable iron peaks (Fe). The increased and uniformly distributed ceria nanoparticles, which serve as effective reinforcements, along with greater degradation temperatures obtained for the 1.5 wt% ceria loading (Figure 6), improved the mechanical and thermal properties of the coating, leading to improved wear resistance and subsequently increasing the wear life of the coating. The SEM images of the wear track at sliding speeds of 0.4 and 0.5 m/s revealed that the primary wear mechanism is a combination of both abrasive and adhesive. To investigate whether a higher ceria content could further improve the wear resistance, PEEK/3 wt% ceria nanocomposite coatings were tested under operating conditions of 70 N and 0.5 m/s. As shown in Figure 15, the PEEK/3 wt% ceria loaded coating did not fail even after 10,000 cycles, demonstrating similar tribological performance to the 1.5 wt% ceria coating. This was confirmed by the EDS analysis and the relatively low wear track depth of ~32 µm (lower than the thickness coating of ~100 µm).

#### 4.5.3. Accelerated Wear Life Testing

**Figure 16:**
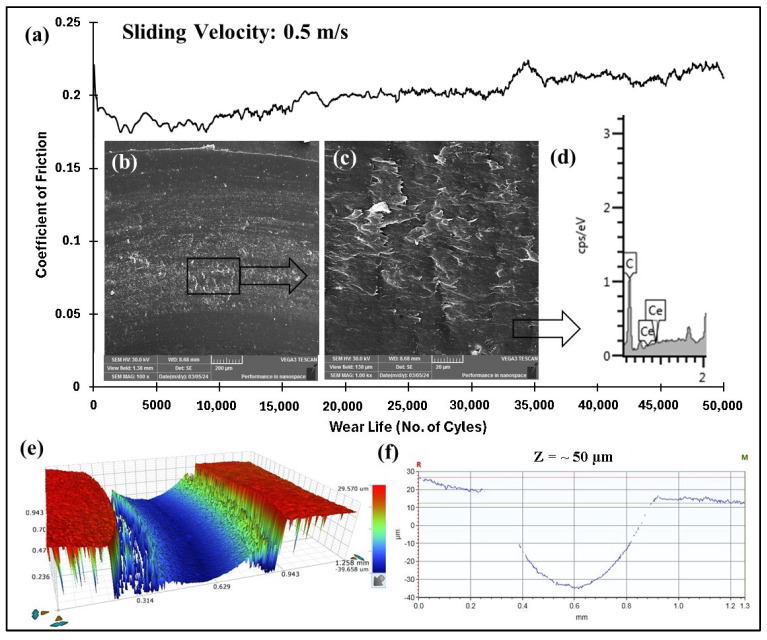
PEEK/1.5 wt% ceria coating at a normal load of 70 N, including the (**a**) frictional graph (**b**,**c**) and SEM images, along with the (**d**) corresponding EDS scan, (**e**) 3D optical scan, and (**f**) 2D optical scan of the wear track for 50,000 cycles.

Long-term wear tests were carried out to assess the tribological evaluation of the PEEK coatings reinforced with 1.5 and 3 wt% ceria. These tests were conducted following their remarkable performance, where neither coating showed any failure even after 10,000 cycles under 70 N and 0.5 m/s. For the extended evaluation, the coatings are subjected to the same operating parameters (70 N and 0.5 m/s), but for over 50,000 cycles, corresponding to 1000 m of sliding distance. The frictional graph of the PEEK/1.5 wt% ceria coating, shown in Figure 16, shows no failure, even after 50,000 cycles, as confirmed by the EDS scan in Figure 15d, demonstrating exceptional wear resistance. The wear track depth also measures ~50 µm, highlighting the coating’s effectiveness in protecting metallic components under severe conditions. This enhanced performance for the 1.5 wt% ceria loading can be attributed to the uniform dispersion of nanoparticles within the PEEK matrix. At this optimal concentration, the ceria nanoparticles effectively interact with the polymer chains of the PEEK matrix, restricting their motion and providing a bridging effect that facilitates better load transfer across the matrix. This uniform reinforcement helps distribute the applied stresses more evenly, reducing localized deformation and improving wear resistance. For an increment in the sliding distance from 189 m to 1000 m (around 430%), there was only around a 79% increment in the wear track depth, which shows the excellent wear resistance property of the coating. The occurrence of plastic deformation and the overlapping of the wear debris can be seen on the wear track, as shown in Figure 16c, which are the characteristics of surface fatigue mechanisms that occur due to the continuous cyclic stresses experienced by the coating at such a high speed and load.

**Figure 17:**
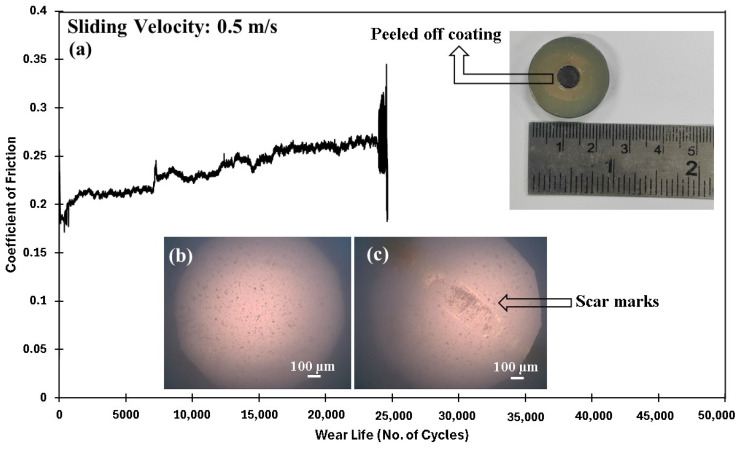
PEEK/3wt% ceria coating at a normal load of 70 N, including (**a**) the frictional graph, along with the peeled-off coating with the (**b**) ball image before and (**c**) after testing with acetone cleaning.

**Figure 18:**
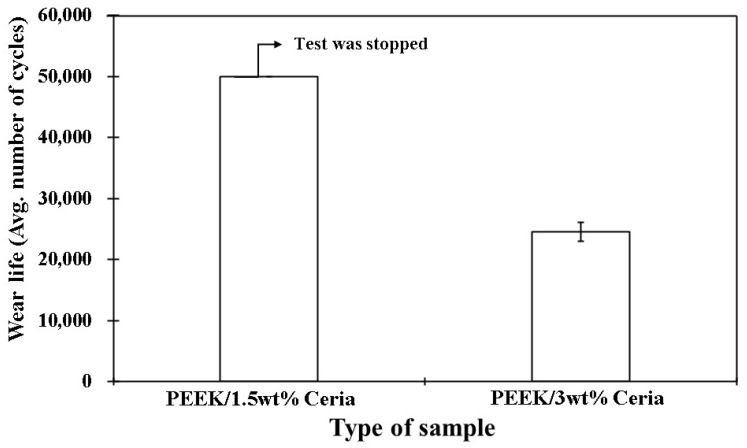
Comparative wear life study of the nanocomposite coatings at a normal load of 70 N for 50,000 cycles at a sliding velocity of 0.5 m/s.

As shown in Figure 17, the 3 wt% loaded ceria coating fails under the same experimental conditions (70 N and 0.5 m/s) when subjected to a prolonged duration of 50,000 cycles (1000 m). The wear life of the coating is ~26,000 cycles. This failure observed in the coating with 3 wt% ceria nanoparticles can be attributed to particle agglomeration (Figure 5c) and the formation of a distinct two-phase structure, namely, a softer phase consisting of the polymer matrix, and a harder phase comprising ceria nanoparticle agglomerates. These agglomerates weaken the bonding interface and lower the bridging capability between polymer chains, thereby reducing the effective load transfer during tribological testing. Consequently, regions dominated by the softer polymer phase act as weak points, facilitating the initiation of coating failure/delamination. Table 1 summarizes the tribological performance at a constant load with varying sliding speeds for different coating compositions. The comparative wear life of PEEK/1.5 wt% and PEEK/3 wt% ceria coating at 70 N, 0.5 m/s for 50,000 cycles is shown in Figure 18.

#### 4.5.4. Effect of Normal Load on the Tribological Performance of PEEK/1.5 wt% Ceria Nanocomposite Coatings

**Figure 19:**
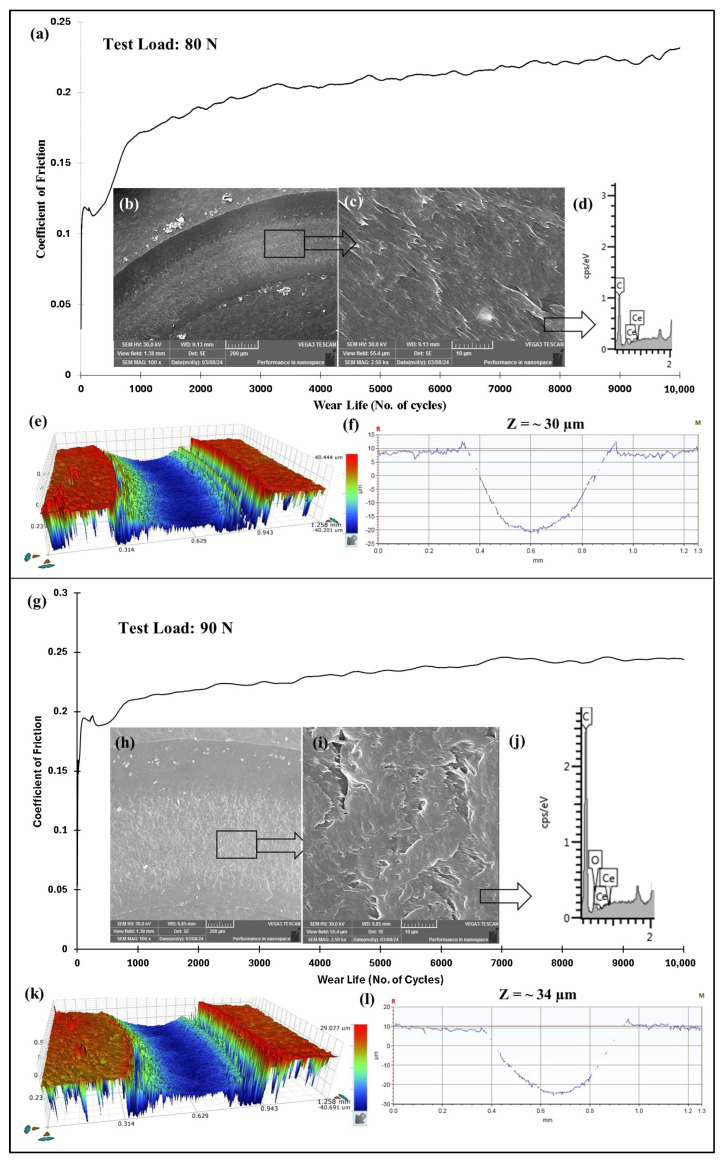
(**a**,**g**) Frictional graphs of PEEK/1.5 wt% ceria coatings, along with the (**b**,**c**,**h**,**i**) SEM images of the wear track with the corresponding (**d**,**j**) EDS scan, (**e**,**k**) 3D profile, and (**f**,**l**) the wear track depth images at varying normal loads for 10,000 cycles at 0.4 m/s.

**Figure 20:**
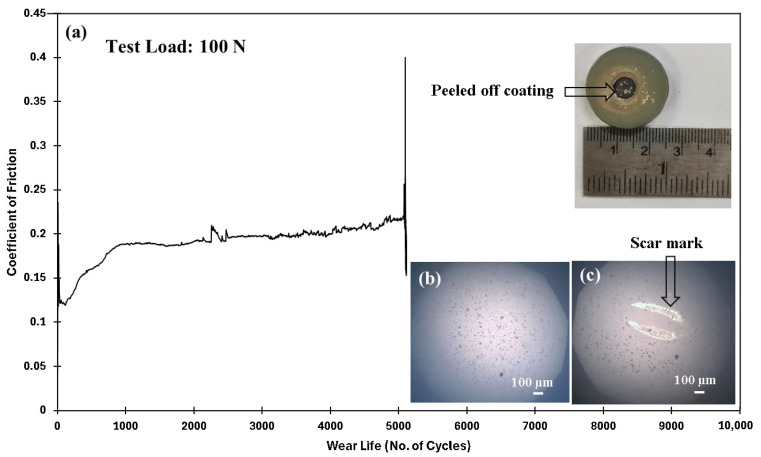
PEEK/1.5 wt% ceria coating at a sliding velocity of 0.4 m/s, including the (**a**) frictional graph, along with the peeled-off coating with the (**b**) ball image before and (**c**) after testing with acetone cleaning.

**Figure 21:**
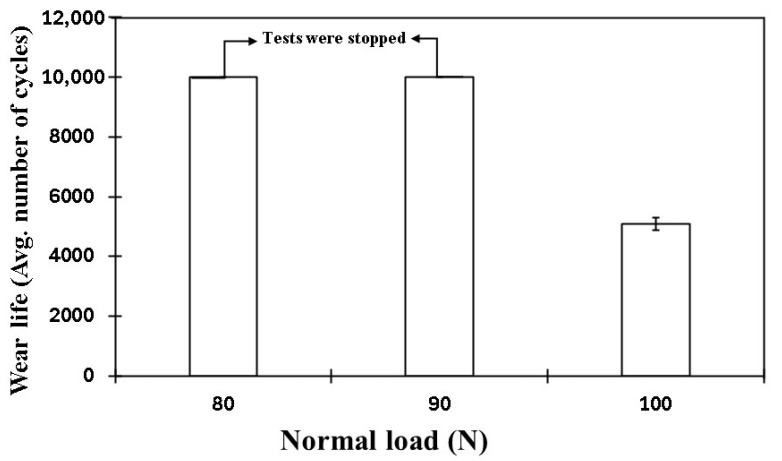
Comparative wear life study of PEEK/1.5 wt% ceria coatings at a sliding velocity of 0.4 m/s for 10,000 cycles at different normal loads.

Following earlier evaluations, it was concluded that the PEEK nanocomposite coating reinforced with 1.5 wt% ceria exhibited exceptional wear resistance at a sliding speed of 0.5 m/s under a load of 70 N. To further explore the tribological performance of this optimized coating, additional tests were conducted at higher loads of 80, 90, and 100, while maintaining a constant sliding speed of 0.4 m/s. As shown in Figure 19, the results include the frictional graph, SEM images of the wear track, EDS scans, and both 2D and 3D profilometric scans of the wear track under normal loads of 80 N and 90 N at 0.4 m/s. The coating demonstrated a remarkable performance, showing no signs of failure, even after 10,000 cycles. The wear track depths measured at ~30 µm and ~34 µm, confirming its excellent wear resistance. The SEM images of the wear track suggest that the primary wear mechanism is abrasive wear due to plastic deformation. Additionally, the EDS scan of the wear track further confirms the non-failure of the coating. However, when the normal load reaches 100 N, the coating fails at ~5100 cycles. This is attributed to a significant rise in contact pressures, causing a localized temperature increase that leads to the coating’s complete degradation, as shown in Figure 20. Figure 21 compares the wear life of the coating under varying loads.

#### 4.5.5. Accelerated Wear Life Testing of PEEK/1.5 wt% Ceria

**Figure 22:**
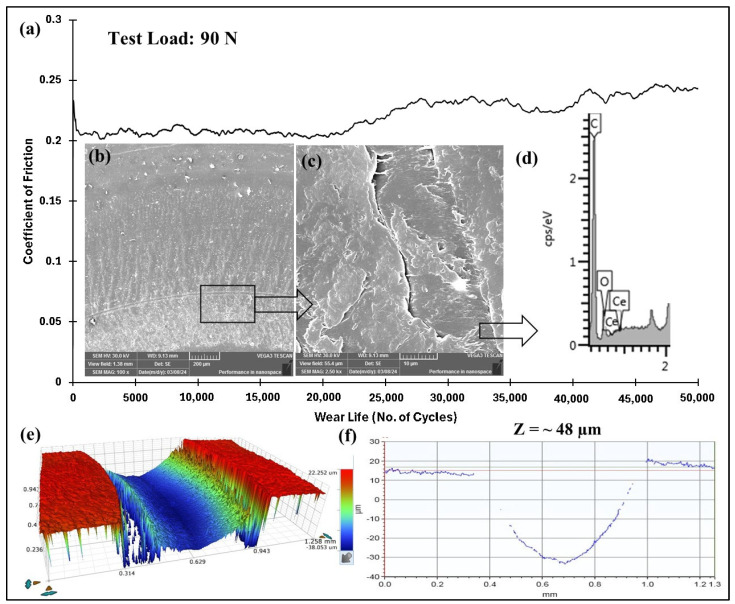
PEEK/1.5 wt% ceria coating at a normal load of 90 N, including the (**a**) frictional graph and (**b**,**c**) SEM images, along with the (**d**) corresponding EDS scan and (**e**) 3D optical scan of the wear track and the (**f**) 2D optical scan of the wear track for 50,000 cycles.

Long-term wear tests were conducted to assess the tribological properties of the optimized PEEK/1.5 wt% ceria nanocomposite coating. These tests were performed under operating conditions of 90 N and 0.4 m/s for 50,000 cycles (1000 m). The frictional graph in Figure 22 demonstrates that the coating does not fail until 50,000 cycles. The wear track’s SEM images confirm the wear mechanism, which was adhesive wear coupled with plastic deformation. The coating’s structural integrity is further confirmed by the EDS scan and the wear track depth, which was found to be ~48 µm. To provide a comparative analysis, Table 2 presents the optimized PEEK + 1.5 wt% ceria coating’s tribological performance at a constant speed with varying loads and cycles.

The performance of PEEK coatings with various reinforcements, as reported in the literature, is summarized in Table 3 and compared with the nanocomposite coating developed in this study. It is evident that the nanocomposite coatings developed here demonstrate enhanced wear resistance, as shown by their lower specific wear rate compared to the results from previous research.

**Table 3:** Comparison of results obtained in this study to past work.

Ref.	Filler	Conc. (wt%)	Substrate	Counterface	Speed (m/s)	Load (N)	Distance (m)	Wear Track Radius (mm)	Thickness (µm)	COF	Sp. Wear Rate (10^−6^ mm^3^/Nm)
[21]	Teflon (PTFE)	3	Stainless steel	100Cr6 ball	NA	10	697	3	200	0.133	38
[26]	Silicon dioxide (SiO_2_)	10	Plain C steel	Steel pin	0.13	11	1000	NA	150	0.56	40
[52]	Carbon fiber (CF)	10	Stainless steel	Ceramic ball	0.2	7	1512	NA	150–200	0.4	75
[23]	Silicon carbide (SiC)	7	Aluminium	100Cr6 ball	1.4	9	2000	NA	40	0.27	20
[53]	Hexagonal boron nitride (h-BN)	1.5	Low-carbon steel	Steel ball	0.1	25	1000	8	200	0.27	20
[54]	Alumina (Al_2_O_3_)	1.5	Titanium alloy	Alumina ball	0.07	5	1000	6	120	0.29	1.9
[55]	Titanium nitride (TiN)	1.3	Titanium alloy	Alumina ball	0.04	5	2000	3	120	0.3	1.1
*	Cerium dioxide (CeO_2_)	1.5	Mild steel	Alumina ball	0.4	90	1000	3	100	0.22	2.042
					0.5	70				0.201	2.692

* Current Research.

Thomasz et al. [54] deposited Al_2_O_3_/PEEK composite coatings on a titanium alloy via the electrophoretic technique. Tribological tests were conducted by a ball-on-disc configuration at a normal load of 5 N and a sliding velocity of 0.07 m/s for 2000 m. The specific wear rate obtained for the composite coatings was 1.9 × 10^−6^ mm^3^/Nm. Similarly, Thomasz et al. [55] deposited TiN/PEEK composite coatings on a titanium alloy via the electrophoretic technique, and similar tribological tests were conducted by a ball-on-disc configuration at a normal load of 5 N and a sliding velocity of 0.04 m/s for 2000 m. The specific wear rate obtained for the composite coatings was 1.1 × 10^−6^ mm^3^/Nm. In comparison to the above-mentioned experiments, the developed coatings in the present study were able to sustain loads of 70 N and 90 N, corresponding to sliding velocities of 0.5 m/s and 0.4 m/s, and exhibiting specific wear rates of 2.692 × 10^−6^ mm^3^/Nm and 2.042 × 10^−6^ mm^3^/Nm, respectively. An increment in the sliding speed by almost tenfold and an increment in the load by a factor of twenty has only resulted in an increase in the specific wear rate of approx. twofold. Coatings were subjected to increased mechanical stresses and frictional forces at these higher loads and velocities, which can accelerate the wear. Nevertheless, the developed coatings held up well under these harsh experimental conditions, indicating better material strength, cohesion, and substrate adherence. As a result, the engineered coatings in this study are well-suited for applications that need wear resistance throughout a broad range of loads and speeds, in contrast to the low-load, low-speed objective attained by the researchers in the previously described studies.

### 4.6. Effect of Different Counterfaces on the Tribological Performance of the Optimized PEEK/1.5 wt% Ceria Nanocomposite Coatings

The tribological performances of PEEK/1.5 wt% ceria nanocomposite coatings are evaluated against different counterface materials, namely, 440C hardened steel (SS), tungsten carbide (WC), silicon nitride (Si_3_N_4_), and alumina (Al_2_O_3_), at a normal load of 90 N, 0.4 m/s, for 10,000 cycles. The surface roughness, coefficient of friction, and wear track depth of each counterface are reported in Table 4.

**Figure 23:**
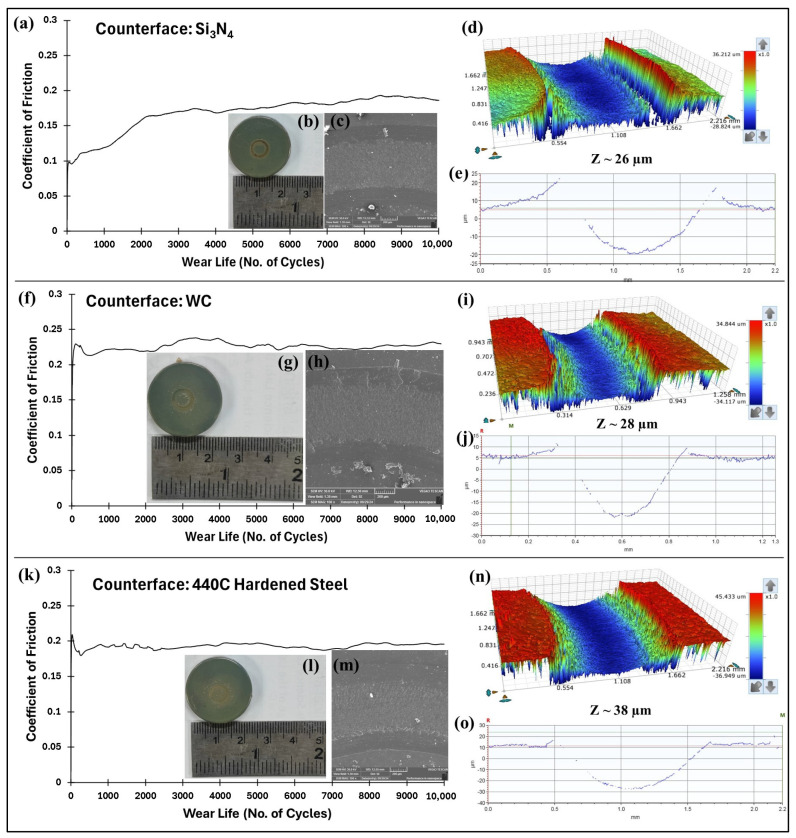
Comparison of the (**a**,**f**,**k**) frictional graph of PEEK/1.5 wt% ceria nanocomposite coatings, along with (**b**,**g**,**l**) wear track photographs and (**c**,**h**,**m**) SEM images with the corresponding (**d**,**i**,**n**) 3D and (**e**,**j**,**o**) 2D profile images of the wear track at a normal load of 90 N at 0.4 m/s for 10,000 cycles.

The experimental results showed that the PEEK/1.5 wt% ceria nanocomposite coating exhibited consistent tribological performance across all tested counterface materials, as can be observed from Figure 18 and Figure 23. The minimal variation in wear depth and friction across different counterface materials highlights its robustness and versatility. This consistent performance suggests the excellent adaptability of the coating under varied tribological conditions, making it highly promising for real-world applications, where counterface materials can vary significantly. Such reliability across alumina, steel, silicon nitride, and tungsten carbide demonstrates the coating’s potential for broad industrial use, particularly in systems where interchangeable or multi-material contact surfaces are common.

### 4.7. Analysis of UV Radiation Tests on the PEEK Coatings

This study performs a comparative analysis to evaluate the effect of UV exposure on pristine PEEK and the optimized PEEK/1.5 wt% ceria nanocomposite coating.

#### 4.7.1. FTIR Analysis of the PEEK and PEEK/1.5 wt% Ceria Nanocomposite Coating Exposed to UV Radiation

**Figure 24:**
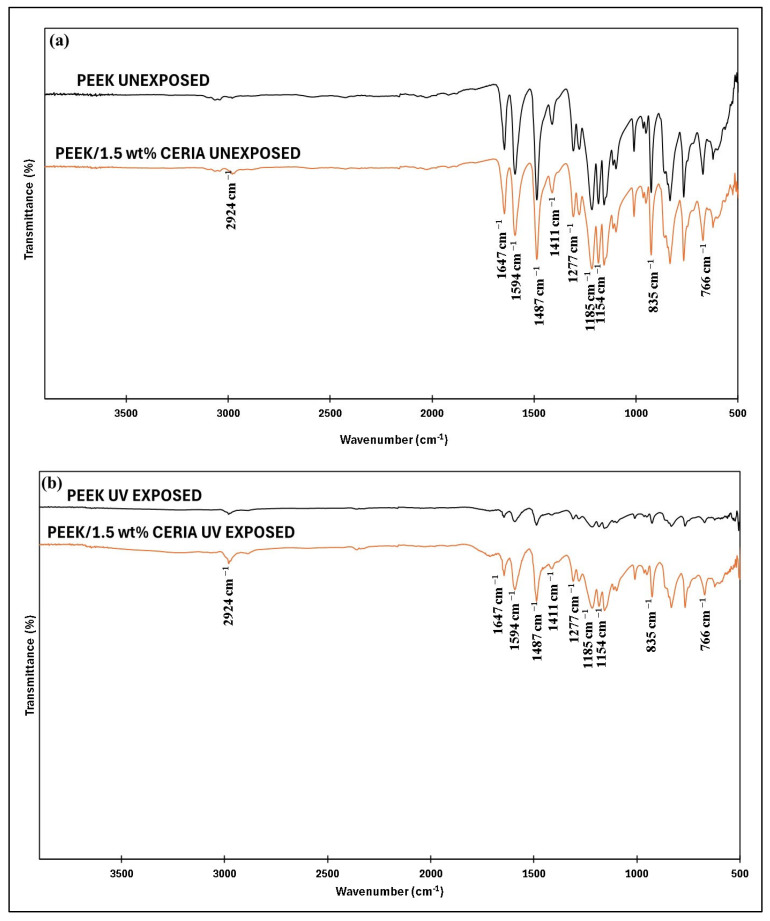
FTIR spectra of the pristine PEEK and PEEK/1.5 wt% ceria coatings (**a**) unexposed and (**b**) exposed to UV radiation.

The FTIR wide spectra from 4000 to 500 cm^−1^ for pristine PEEK and PEEK/1.5 wt% ceria nanocomposite coatings, unexposed and exposed to UV radiation, are shown in Figure 24a and b, respectively. As can be observed from Figure 24a, the FTIR spectra of the unexposed pristine PEEK exhibited a C=C stretching at 1647 cm^−1^ and a vibration of the skeletal ring at 1594 cm^−1^, 1487 cm^−1^, and 1411 cm^−1^, respectively [56,57,58]. The peak at around 1154 cm^−1^ corresponds to C-O-C stretching [59]. The vibration bands of the diphenyl ether group’s asymmetric stretching were observed at 1277 cm^−1^ and 1185 cm^−1^ [60]. The absorbance observed at 835 cm^−1^ and 766 cm^−1^ correlates with the C-H out-of-plane bending vibrations [61]. Furthermore, the FTIR spectrum of the unexposed PEEK/1.5 wt% ceria consisted of the signature peak of ceria at 2924 cm^−1^, corresponding to −CH_3_ and −CH_2_ asymmetric stretching [62], in addition to all the signature peaks of PEEK. However, after exposing the coatings to UV radiation (Figure 24b), the intensity of the peaks of PEEK matrix drastically reduced, suggesting a significant degradation of PEEK after UV exposure [63,64]. Furthermore, the FTIR spectrum of the UV-exposed PEEK/1.5 wt% ceria composite coating showed all the signature peaks of PEEK and ceria at similar positions as the unexposed sample. Even though the peak intensities exhibited by the UV-exposed PEEK/1.5 wt% ceria composite coating were reduced, their intensities were much higher compared to the UV-exposed pristine PEEK coatings, suggesting that the composite coating showed better resistance to degradation on UV exposure [65,66,67]. This was because the ceria nanoparticles exhibit a fluorite-type lattice with a high density of oxygen vacancies, which facilitates the strong absorption of UV light, particularly in the UVA (320–400 nm) and UVB (280–320 nm) regions, by enabling electronic transitions between Ce^4+^ and Ce^3+^ oxidation states [36]. This absorption reduces the penetration of harmful UV radiation into the polymer matrix, thereby preventing photodegradation of the host material. In addition to their UV-shielding effect, ceria nanoparticles serve as potent antioxidants. They exhibit reversible redox behavior, switching between Ce^4+^ and Ce^3+^ states in response to oxidative environments [32]. This redox cycling enables ceria to neutralize free radicals and reactive oxygen species, which are commonly generated under UV exposure and are responsible for polymer chain scission and oxidation [68].

#### 4.7.2. Tribological Performance of Pristine PEEK and Optimized PEEK/1.5 wt% Ceria Nanocomposite Coatings Before and After Exposure to UV Radiation

**Figure 25:**
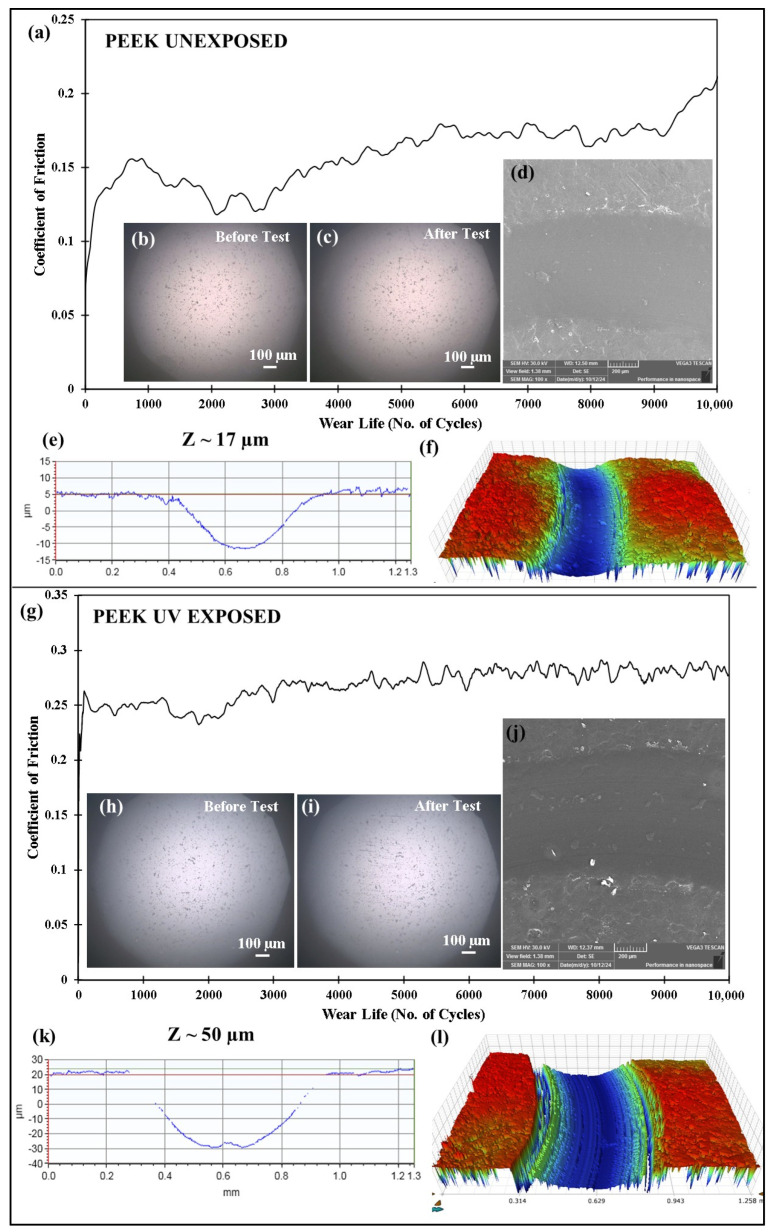
Comparison of the (**a**,**g**) frictional graph of PEEK coatings, along with the (**b**,**h**) before test ball and (**c**,**i**) after test ball images, with the (**d**,**j**) SEM images and the corresponding (**e**,**k**) 2D profile and (**f**,**l**) 3D profile wear track depth images at a normal load of 50 N at 0.1 m/s for 10,000 cycles.

**Figure 26:**
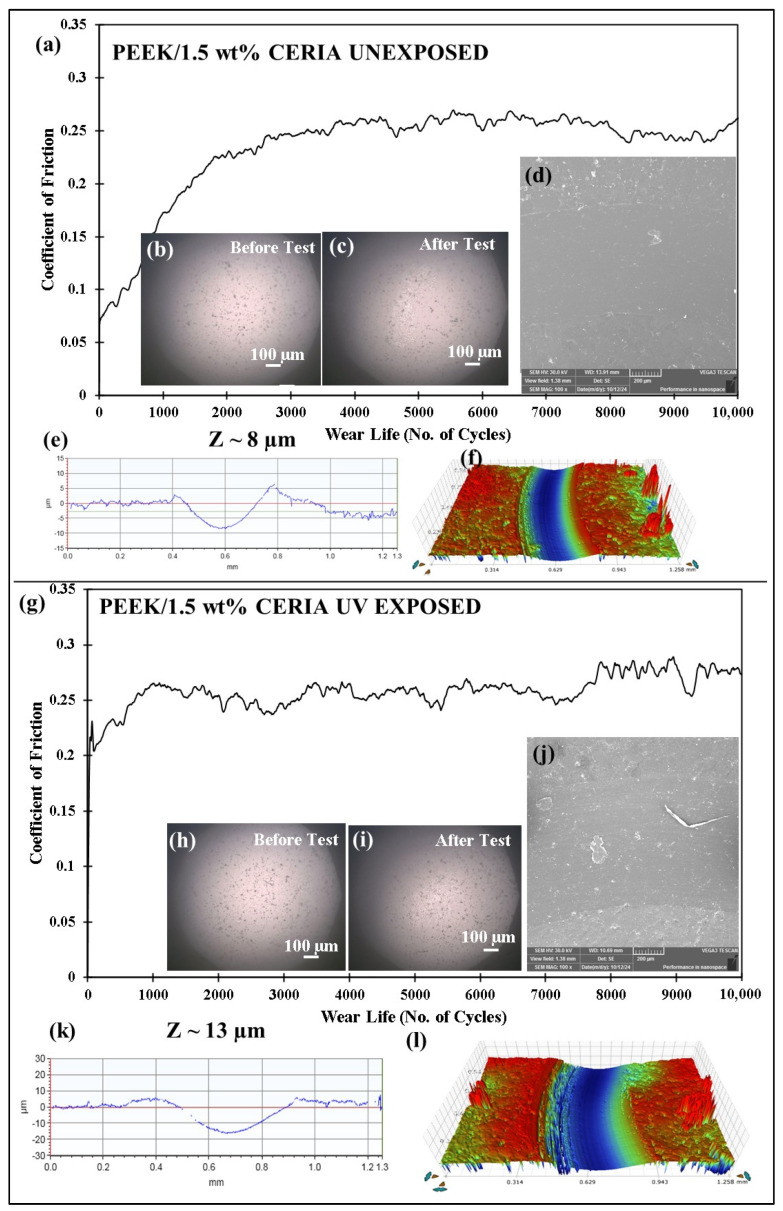
Comparison of the (**a**,**g**) frictional graph of the PEEK/1.5 wt% ceria coatings, along with the (**b**,**h**) before test ball and (**c**,**i**) after test ball images, with the (**d**,**j**) SEM images and the corresponding (**e**,**k**) 2D profile and (**f**,**l**) 3D profile wear track depth images at a normal load of 50 N at 0.1 m/s for 10,000 cycles.

Wear tests are conducted on pristine PEEK and PEEK/1.5 wt% ceria composite coatings before and after UV exposure to evaluate their wear performance. The tribological tests are performed at 50 N load and 0.1 m/s sliding speed for 10,000 cycles, corresponding to a total sliding distance of 189 m. The results indicate that the unexposed pristine PEEK coating exhibited a moderate wear depth of ~17 µm (Figure 25e), as compared to a significantly higher wear depth of ~50 µm (Figure 25k) after UV exposure. This is attributed to the degradation of the PEEK polymer structure due to exposure to the UV radiation, as suggested by the FTIR spectrum (Figure 24b). The PEEK/1.5 wt% ceria nanocomposite coatings show significantly better wear resistance than pristine PEEK, exhibiting much lower wear depth in both UV-unexposed and UV-exposed conditions. The unexposed nanocomposite coating exhibited a wear depth of only ~8 µm (Figure 26e), and the UV-exposed composite coating demonstrated a wear depth of ~13 µm only (Figure 26k). The improved performance of the composite coating is primarily due to the enhancement of PEEK’s overall mechanical and thermal properties, as well as its UV resistance, achieved through the addition of ceria nanoparticles. This highlights the advantages of incorporating ceria to improve the wear durability and UV stability of PEEK.

## 5. Conclusions

This study offers an in-depth analysis of the mechanical and tribological properties of pristine PEEK and PEEK nanocomposite coatings reinforced with cerium dioxide (ceria/CeO_2_) under different operating conditions. The key findings of this research are summarized below:

- At a normal load of 70 N and a sliding speed of 0.3 m/s, the pristine PEEK coating exhibited a wear life of approximately 7500 cycles before failure.
- The coating’s wear life was significantly increased by the incorporation of ceria nanofillers; the ideal filler loading was found to be 1.5 wt%, resulting in a wear life of over 50,000 cycles at a normal load of 70 N and a linear speed of 0.5 m/s.
- Among the tested concentrations, the 1.5 wt% ceria also demonstrated optimal performance, recording a wear life of over 50,000 cycles when tested at a load of 90 N, with a linear speed of 0.4 m/s.
- A lower ceria concentration provided an insufficient bridging effect, and the formation of agglomerations were found to be the leading reasons for the premature failure of the 0.5 and 3 wt% loadings, respectively.
- The counterface materials had minimal impact on the tribological performance of the PEEK/1.5 wt% ceria nanocomposite coating, indicating that the coating effectively maintained its wear resistance and stability regardless of the counterface in contact.
- This study also confirmed that UV exposure degrades pristine PEEK’s wear performance, while the addition of ceria nanoparticles was found to significantly enhance UV resistance, thereby improving the mechanical and tribological properties of the composite coating.

## Figures and Tables

**Table 1:** Comparative summary of tribological performance at constant load with varying sliding speeds and cycles.

Coating	Load (N)/Speed (m/s)/Sliding Distance (Cycles)	Wear Track Depth (µm)	Coefficient of Friction (COF)	Outcome
Pristine PEEK	70/0.1/10,000	29	0.31	No Failure
	70/0.2/10,000	70	0.35	No Failure
	70/0.3/10,000	N/A	N/A	Failed (~7500 cycles)
PEEK/0.5 wt% CeO_2_	70/0.3/10,000	30	0.23	No Failure
	70/0.4/10,000	N/A	N/A	Failed (~3100 cycles)
PEEK/1.5 wt% CeO_2_	70/0.4/10,000	26	0.22	No Failure
	70/0.5/10,000	28	0.19	No Failure
	70/0.5/50,000	50	0.2	No Failure
PEEK/3 wt% CeO_2_	70/0.5/10,000	32	0.21	No Failure
	70/0.5/50,000	N/A	N/A	Failed (~26,000 cycles)

**Table 2:** Overview of the tribological performance of optimized PEEK/1.5 wt% ceria coating at a constant speed with varying loads and cycles.

Coating	Load (N)/Speed (m/s)/Sliding Distance (Cycles)	Wear Track Depth (µm)	Coefficient of Friction (COF)	Outcome
PEEK/1.5 wt% CeO_2_	80/0.4/10,000	30	0.22	No Failure
	90/0.4/10,000	34	0.24	No Failure
	100/0.4/10,000	N/A	N/A	Failed (~5100 cycles)
	90/0.4/50,000	48	0.204	No Failure

**Table 4:** Measurements of the roughness and water contact angles of the counterfaces.

Material	Roughness (Ra/µm)	Coefficient of Friction	Wear Track Depth (µm)
440C hardened steel	3.141	0.2263	38
Alumina (Al_2_O_3_)	3.187	0.2307	34
Tungsten carbide (WC)	3.241	0.193	28
Silicon nitride (Si_3_N_4_)	3.287	0.1693	26

## Data Availability

The original contributions presented in this study are included in the article. Further inquiries can be directed to the corresponding author.
